# Improvement project in higher education institutions: A BPEP-based model

**DOI:** 10.1371/journal.pone.0227353

**Published:** 2020-01-03

**Authors:** Marco Maciel-Monteon, Jorge Limon-Romero, Carlos Gastelum-Acosta, Yolanda Baez-Lopez, Diego Tlapa, Manuel Iván Rodríguez Borbón

**Affiliations:** 1 Facultad de Ingeniería, Arquitectura y Diseño, Universidad Autónoma de Baja California, Ensenada, Baja California, México; 2 Departamento de Ingeniería Industrial y Manufactura, Instituto de Ingeniería y Tecnología, Universidad Autónoma de Ciudad Juárez, Ciudad Juárez, Chihuahua, México; Univerza v Mariboru, SLOVENIA

## Abstract

Improvement projects (IPs) are a fundamental element in any quality management system from any organization. In Higher Education Institutions (HEIs), IPs are constantly implemented to maintain excellence in academic and administrative processes. In this study, we propose a model for IP implementation that is based on the Baldrige Performance Excellence Program (BPEP). As a part of the model, we propose a series of research hypotheses to be tested. The data used to test the hypotheses were gathered from a questionnaire that was developed after an extensive literature review. The survey was administered to Mexican public HEIs, and more than 700 responses were collected. The data were assessed in terms of convergent and discriminant validity, obtaining satisfactory results. To test the proposed relationships between the model constructs, we utilized Structural Equation Modeling (SEM) using the software IBM SPSS Amos. The analysis confirmed the statistical validity of both the model and the hypotheses. In conclusion, our model for IP implementation is a useful tool for HEIs that seek to attain excellence in their processes through IPs.

## Introduction

Global competition forces organizations to preserve high quality in their products and services to ensure customer satisfaction. From a similar perspective, Higher Education Institutions (HEIs) must guarantee that their programs and processes allow students to become professionals with skills and competencies that current globalized environments demand. Moreover, according to Ah-Teck and Starr [1], quality educational systems are the key to the world´s economic development, the quality of a country’s higher education is one of the fundamental pillars of its development, many countries have started to take government action in this respect [2].

In order to improve, many HEIs incorporate quality management strategies[3]–such as Six Sigma (SS), Total Quality Management (TQM), Lean Six Sigma (LSS), and Kaizen–that use Improvement Projects (IPs) to increase quality and productivity in organizational processes. Regardless of the philosophy and goals that each strategy promotes, they all rely on IPs to obtain the desired results [4]. According to Juran, Gryna, and Bingham [5], quality improvement implies solving the chronic problems of processes; moreover, improvements occur project after project, not overnight. Similarly, to Gonzalez Aleu and Van Aken [6], IPs are systematic processes carried out by teams of people to improve the processes and systems of an organization with minimum investment of capital in a relatively short time. IPs are usually carried out in methodologies such as the lean manufacturing of kaizen events, the DMAIC of SS projects, in hybrid approaches of LSS projects, and in the Plan-Do-Check-Act (PDCA) technique for continuous improvement.

This research uses the Baldrige model of excellence as the structure for an IP implementation model. In the education field, the Baldrige model is internationally recognized as a management system that helps understand how education institutions work and highlights strengths and weaknesses in processes, as well as opportunities for improvement. The Baldrige Performance Excellence Program (BPEP) provides an excellent vision to diagnose and define priorities for improvement, shape a customer-oriented culture, optimize resources, and preserve the desired outcomes. According to Ruben, Russ, Smulowitz, and Connaughton [7], the Baldrige model of excellence has many advantages for HEIs. First, it applies recognized standards of organizational excellence and can be implemented in an entire organization or in specific departments or academic programs. Second, the BPEP can be adapted to academic processes, student services, and/or business units. Third, the Baldrige model creates reference measures, provides a framework for sharing effective practices, expands participation in leadership and problem solving, and complements new accreditation models. Finally, the BPEP is a useful tool to develop and manage quality systems [8], since it seeks to respond to the needs of HEIs by combining innovation and results through good practices adopted in administrative processes. As previously mentioned, the proposed model for developing IPs under the BPEP structure, was validated with information obtained from Mexican public HEIs, which cover a little more than 70% of the enrollment in higher education in this country.

## Research gap

The HEIs, in addition to the globalized competition they face daily, have to locally face the demands of the productive and social sectors with respect to the relevance of their educational offerings. In the case of public HEIs, they are subject to the economic restrictions of state and federal governments, which are the main sources of their operating budgets [9]. Likewise, there is an internal requirement on the part of students, parents and employers to adjust the quality of education to the labor market [10]. Therefore, an important challenge for HEIs is the strengthening of their administrative structures through incorporating quality assurance systems to be productive and efficient with the use of resources, which may be scarce in some cases. The realization of IPs is a very useful tool for the manufacturing sector as well as for the service sector; thus, in the education sector, it should not be an exception. According to the literature, IPs have been carried out in HEIs around the world with the help of different methodologies and not under a single structure. With regard to BPEP as a framework used by organizations to guide the efforts of continuous improvement actions, as mentioned above, there are several articles in the literature that highlight their importance in HEIs; however, the literature review indicates the absence of a model that represents the way in which the critical factors of BPEP interact during the realization of IPs in HEIs. Therefore, this study proposes and validates a model based on structural equation modeling (SEM) to quantitatively represent these interactions; this model is intended to function as a guide to increase the probability of obtaining the expected results with the realization of such projects in HEIs.

## Literature review

Over the years, the quality of goods produced in the manufacturing sector has become a critical point that must be controlled. IPs are implemented to improve processes and to lead organizations or companies into a cycle of continuous improvement, seeking from the beginning not only to maintain but to improve the quality of their processes, products or services. For example, [11] demonstrated in their study the empirical application of DMAIC projects to reduce the failure rate in high voltage tests of one of the most critical products. On the other hand, the main lean practices that the manufacturing industries implement are those related to waste disposal or quality improvement [12]. Thus, projects with different structures are conducted but are focused on improving productivity and customer satisfaction in industries [13–16].

The previous paragraph mentions the realization of different IPs in the manufacturing industry. Likewise, in recent years, it has been noted how organizations belonging to the service sector have joined the continuous search for the improvement of the quality of services offered to their customers, applying methodologies or strategies that were initially exclusive to the manufacturing sector. An example is the government sector, where [17] present a theoretical and practical guide that can serve as a basis for local governments seeking to implement kaizen in administrative management. Similarly, IPs are carried out in financial institutions to improve service quality and guarantee customer satisfaction [18]. They have also been mentioned in sectors such as aquaculture [19] and irrigation [20] to make better use of resources. Another sector in which the application of these strategies has become popular is related to the administration of hospitals, where it has been shown that the IPs that have been implemented and tested have shown positive results is this area [21–24]. There are even hospitals that have undergone a whole transformation by adopting Lean HealthCare as part of their policies to achieve continuous improvement of their processes. According to Antony et al. [25], the benefits reported in hospitals are as follows: improved operational efficiencies; reduced error rate, waste and operational losses; reduced delay and improved cycle times; improved service quality; positive change in culture, eliminated unnecessary or non-value-added steps in the process; improved customer or patient satisfaction; and reduced operational costs.

The education sector is also interested in achieving continuous improvement in its processes, so they have decided to examine what is being done in other sectors. Continuous quality improvement is attained when organizations apply problem solving and problem mitigation strategies [26]. IPs comprise these strategies, as they encompass systematic improvement actions that can be executed through different methodologies. For instance, IPs are conducted as a part of SS projects for university libraries [27,28] or within LSS programs for planning processes and holding kaizen events [3]. Additionally, IPs have been implemented as a part of university LSS programs [29] and SS projects [30]. The literature and success cases of IPs indicate a recent trend of promoting the aforementioned continuous improvement methodologies—which are typically implemented in other sectors—among HEIs [31,32]. However, a model for IP implementation in the education field has not yet been developed.

### The Baldrige Performance Excellence Program (BPEP)

The BPEP is recognized in the international community as an integral and systematic framework for evaluating excellence in performance and guiding quality improvement efforts [1]. The seven dimensions of the BPEP are strongly interrelated and cover all aspects of an organization or HEI [33]; they can be listed as follows:

- Leadership
- Strategy
- Customers
- Workforce
- Operations
- Measurement, Analysis, and Knowledge Management (MAKM)
- Results

**Fig 1:**
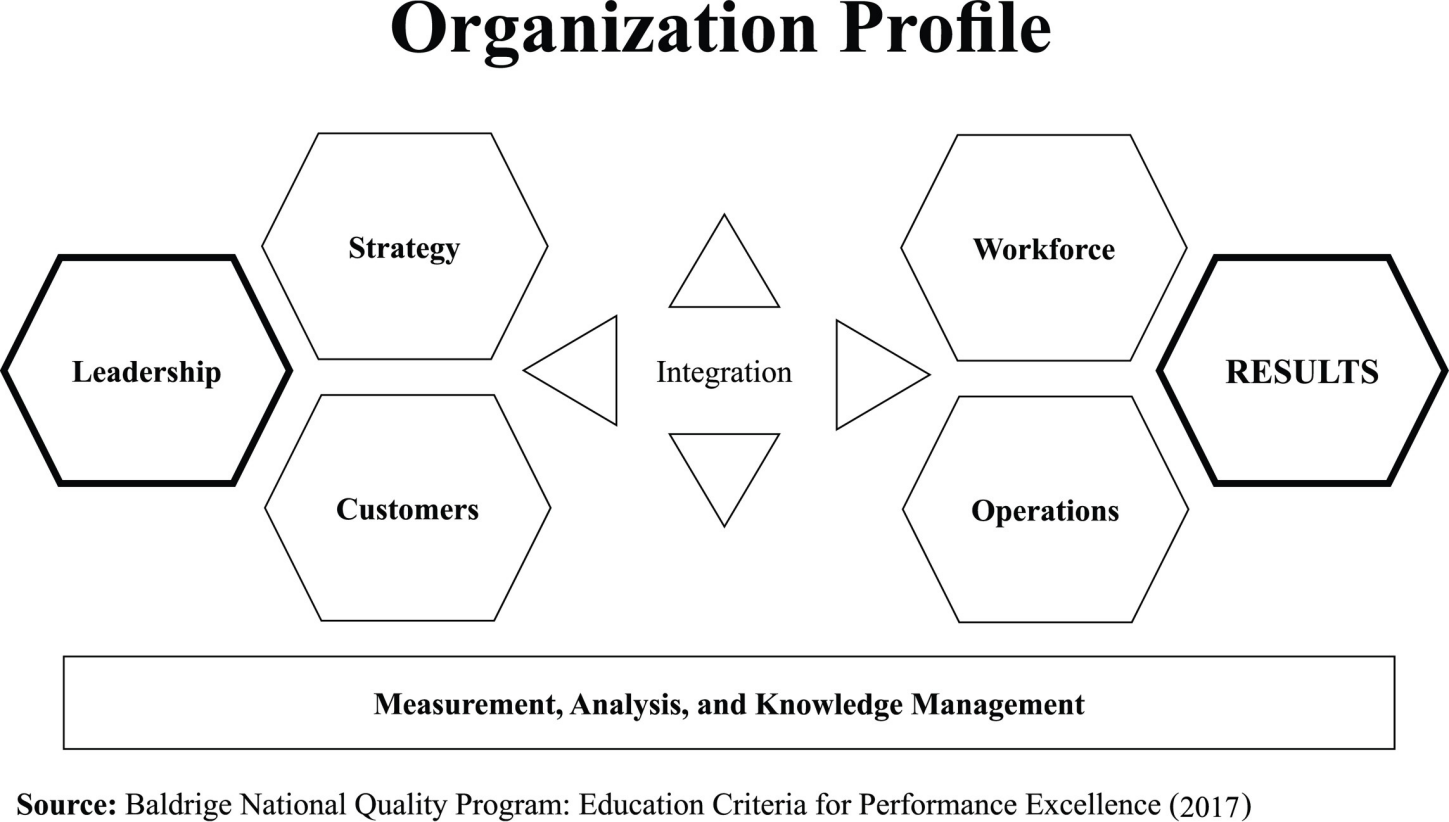
Baldrige Education criteria for the performance excellence model.

The Baldrige Excellence Framework: A Systems Approach to Improving Your Organization's Performance (Education), published by the National Institute of Standards and Technology (NIST) [33], is depicted in Fig 1. BPEP's general theory claims that "leadership drives the system that creates results" and suggests that performance relationships are recursive [34]. In a recursive model, constructs do not have arrows in both directions [35]. As can be seen in Fig 1, the core of the BPEP model is integration, which implies that the model sees all its elements as interrelated components. The horizontal arrowheads in the center of the figure show the critical link between the leadership group–formed by Leadership, Strategy, and Customers–and the results group–composed of Workforce, Operations, and Results. Finally, the vertical arrowheads at the center of the figure point to and from the base of the system, which provides information and feedback on key processes and the organizational environment.

The Leadership dimension of the Baldrige model seeks to evaluate the actions of institutional leaders to attain the strategic goals of education institutions [36]. Leadership is a critical factor in the implementation of quality systems, such as TQM and ISO series, or models of excellence, including the European Framework for Quality Management (EFQM). As for the Strategy dimension, it questions how organizations develop strategic objectives and action plans, implement them, modify them if required, and measure progress [33]. In this sense, authors [36] emphasize on the importance of understanding customer needs to establish appropriate strategies. As regards the Customers dimension of the BPEP model, it involves university students, teachers, and administrative staff for long-term market success. This category listens to the voice of the client (i.e. the student) and uses the information to improve and identify opportunities for innovation [33]. In their work [8], highlight the importance of measuring student satisfaction through indicators and by listening to them to respond to opinions and complaints.

According to the NIST [33] and Badri et al. [8], MAKM examines the organization of a system for the collection, processing, storage, analysis, and distribution of information and knowledge assets in order to support decision-making in operational management and generate better results. On the other hand, the Workforce dimension of the BPEP model emphasizes on the need for human resource planning to attain organizational goals. Moreover, it helps organizations see how they assess the capacity needs of the workforce and how they can create an environment propitious to high performance [33]. For authors [37], workforce enables quality in organizations; the best way to achieve success is to continuously work with, train, and motivate people.

The Operations dimension seeks the efficient and effective management of learning and support processes that create value for stakeholders, evaluators, continuous improvement, and organizational learning [8]. In other words, operations seek to improve operational efficiency to offer value to students and other customers [33]. Finally, the Results dimension of the BPEP model is based on the comparison of an organization with its direct competitors and organizations with similar educational offers and services. The results category covers the areas of teaching and student learning outcomes, workforce performance, and budgetary, financial, and market results [33].

## Research hypotheses and conceptual framework

**Fig 2:**
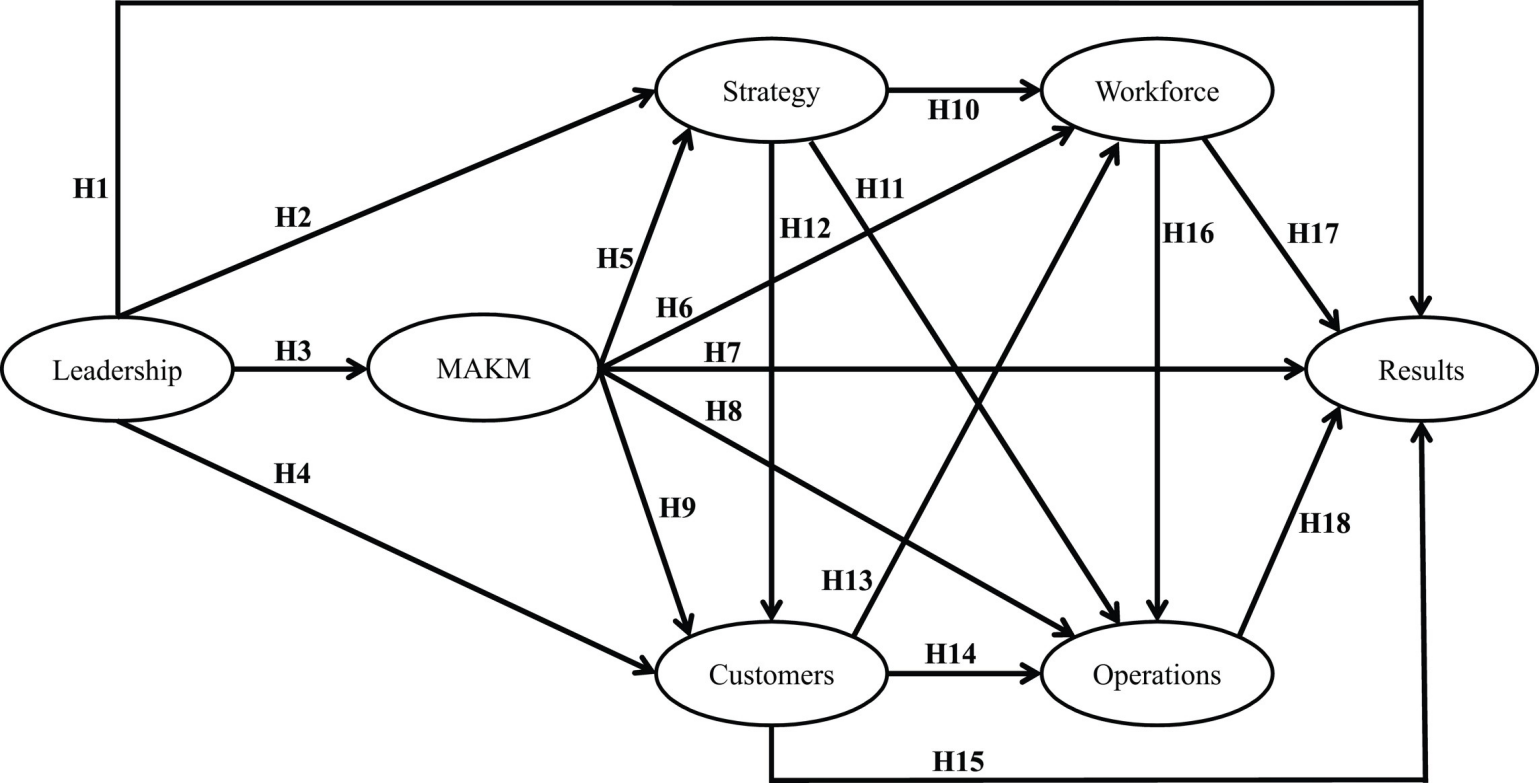
Research model and hypotheses.

The BPEP model has been theoretically validated in the manufacturing and services industries [38,39]. In the following paragraphs we review the works that have reported these validations, as well as relevant literature reported in the education field. Then, we discuss our research hypotheses (Fig 2).

Researchers [40] claim that, in academic environments, leadership plays the role of a quality controller. Similarly, research has found that leadership has a direct relationship with the other dimensions of the BPEP model, including Strategy, MAKM, Customers, and Results [1,8,38,41,42]. In this sense, it is possible to formulate the hypotheses shown in the Table 1.

**Table 1:** Hypotheses considering the effect of leadership on other BPEP dimensions.

Hypotheses	Proposed Relationship
H1	Leadership has a positive effect on Results
H2	Leadership has a positive effect on Strategy
H3	Leadership has a positive effect on MAKM
H4	Leadership has a positive effect on Customers

The MAKM dimension is fundamental for effective organization management, performance, and competitiveness, whereas MAKM indicators ensure that a system is agile and based on facts and knowledge. MAKM also has a direct influence on all the other dimensions of the BPEP model [33], since it provides information and feedback on key processes and the organizational environment [1,8,38,40,42]. Considering these arguments, the next five hypotheses can be proposed as shown in Table 2:

**Table 2:** Hypotheses considering the effect of MAKM on other BPEP dimensions.

Hypotheses	Proposed Relationship
H5	MAKM has a positive effect on Strategy
H6	MAKM has a positive effect on Workforce
H7	MAKM has a positive effect on Results
H8	MAKM has a positive effect on Operations
H9	MAKM has a positive effect on Customers

There is a critical relationship between Strategy and Customers–from the Leadership triad–and Workforce and Operations–from the Results triad [33]. Universities have exhibited their commitment to student satisfaction through mission statements, goals/objectives, marketing strategies, and promotional themes[43]. Moreover, research has found that the four dimensions are interrelated [1,8,38–42]. Following these findings, it is possible to propose the hypotheses shown in the Table 3.

**Table 3:** Hypotheses considering the relationships among Strategy, workforce, operations and customers.

Hypotheses	Proposed Relationship
H10	Strategy has a positive effect on Workforce
H11	Strategy has a positive effect on Operations
H12	Strategy has a positive effect on Customers
H13	Customers have a positive effect on Workforce
H14	Customers have a positive effect on Operations
H16	Workforce has a positive effect on Operations

Since every action leads to a result [33], our model considers the Results dimension as the dependent variable. Moreover, the unidirectional arrows are used to explore the relationships between the BPEP dimensions [1]. According to the literature, workforce and operations have a direct impact on the results of an organization, whereas the remaining dimensions (i.e. leadership, strategy, customers, and MAKM) have a rather indirect effect [8,38–42]. Following this discussion, the last three hypotheses can be formulated as shown in Table 4.

**Table 4:** Hypotheses considering the relationships from customer, workforce and operations on Results.

Hypotheses	Proposed Relationship
H15	Customers have a positive effect on Results
H17	Workforce has a positive effect on Results
H18	Operations have a positive effect on Results

## Research methodology

**Fig 3:**
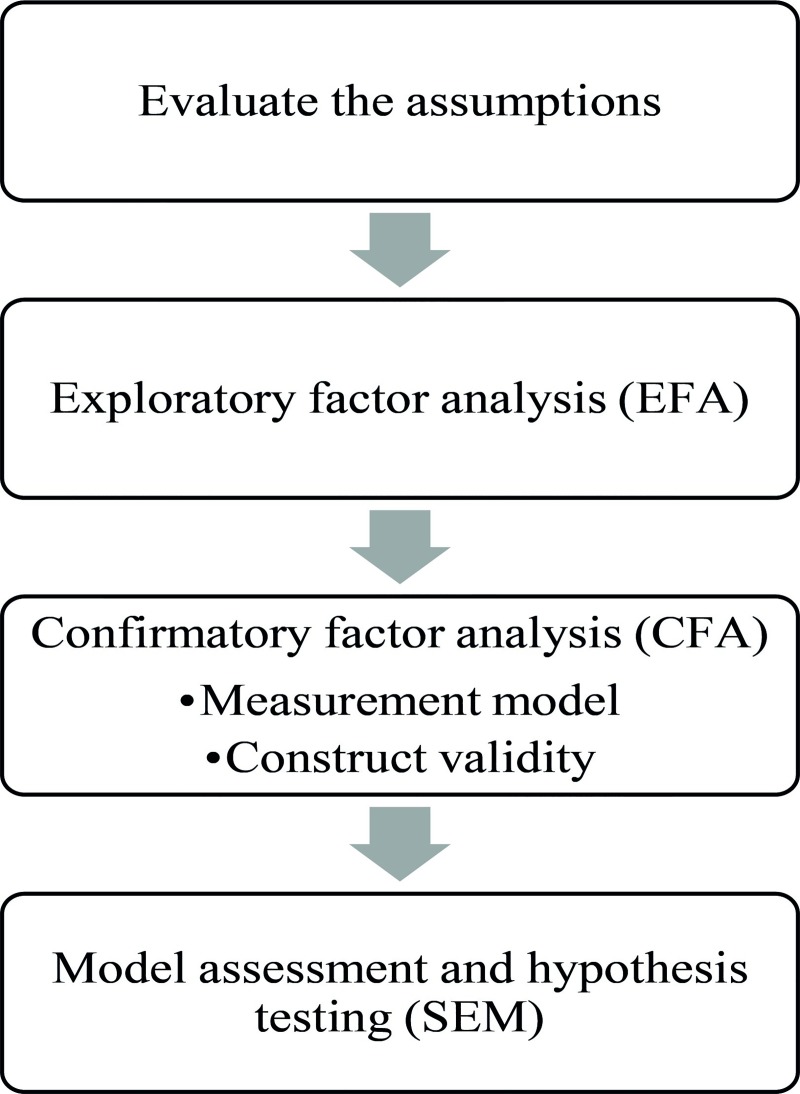
Steps followed to carry out the stages survey validation and model assessment.

This section describes the steps taken to attain our research goals. Our research methodology comprises four main stages: survey development, survey administration, survey validation and model assessment. The first two stages are explained in the following paragraphs in this same section. On the other hand, to carry out the remaining two stages, the steps mentioned below (Fig 3) and whose analyses were performed at each stage are explained in detail in the data analysis and results section that followed.

It is worth mentioning that to verify the hypotheses raised by fitting the structural model, the two phases proposed by Hair et al. [35] were followed, where it is stated that the measurement model must first be validated, which represents the theory showing how measured variables (items) come together to represent constructs and later validate the structural model, which in turn shows how constructs are associated with each other because only when the measurement model is validated and achieves acceptable model fit indices can we turn our attention to testing the proposed structural relationships.

### Survey development

As the research instrument, the survey allowed us to collect the necessary data and test and validate our model. The seven BPEP dimensions were operationalized through survey items that captured the key elements of the BPEP Education Criteria application guide. To develop the instrument, we relied on the works of [3,8,44–49]. The final survey comprised five sections; the first three aimed at introducing the survey, collecting general information on the surveyed HEIs, and analyzing the quality tools used in IP implementation, respectively. On the other hand, the goal of the fourth section was to assess the seven BPEP dimensions during a typical IP implementation process. Finally, the fifth section sought to gather data on the benefits that HEIs by implementing IPs.

### Sampling procedure

To ensure a more comprehensive study, the research was conducted among Mexican public HEIs, which represent more than 70% of national enrollments in higher education [50]. The survey was administered online to quality coordinators and personnel with experience in IP implementation. In total, we collected 743 surveys from 318 public HEIs.

### Ethics statement

The survey contained a cover page stating responses were anonymous and voluntary. By responding to the questions, the subjects agreed to participate in the research. All study participants were informed about the anonymity and confidentiality of their responses; the online platform to request the answers was set to maintain the data anonymous. The research conforms to the provisions of the Declaration of Helsinki (revised in Fortaleza, Brazil 2013) [51], and all ethical guidelines were followed as required for conducting human research, including adherence to the legal requirements of Mexico. This procedure was approved by the Head of the Faculty of Engineering, Architecture and Design of the Autonomous University of Baja California.

## Data analysis and results

According to Hair et al. [35] and Kline [52], four important issues must be considered before the SEM analysis is performed: outliers, univariate and multivariate normality assumptions, and multicollinearity. Table 5 summarizes the analysis results. As can be observed, the basic assumptions of the SEM analysis were not violated after outliers elimination.

**Table 5:** Assumptions results.

Issues	Results	Recommended Values
Outliers	195 significant responses.	Mahalanobis distance, with a level of statistical significance of p <0.001 [52].
Univariate normality	Kurtosis (-0.790, 1.298), Skewness (-1.205,-0.096)	Kurtosis range of ±3 [53], Skewness range of ±2 [54].
Multivariate normality[55,56]	Multivariate kurtosis 245.96, obtained through SPSS Amos® (Arbuckle, 2014).	Value lower than that obtained from the formula p (p + 2), where p is the number of measured variables in the model [57]; the formula yielded a value of 2208.
Multicollinearity	Correlation coefficients lower than the maximum recommended value.	The correlation coefficient between pairs of measured variables must be lower than 0.85 [57].
	Maximum calculated value: 5.8.	Variance inflation factor (VIF) with values lower than 10 [52].

### Results of the Exploratory Factor Analysis (EFA)

To determine the adequacy of the sample, we performed Kaiser Meyer Olkin (KMO) and Bartlett’s sphericity test. Kaiser and Rice [58] recommend KMO values greater than 0.9, whereas the p value from Bartlett’s sphericity test must be lower than 0.01. The values obtained in this research were KMO = 0.98 and p <0.0, and they thus confirmed the feasibility of the factor analysis. After performing the exploratory factor analysis (EFA), seven constructs and 46 measured variables with significant loadings were consolidated. The results are displayed in Table 6.

**Table 6:** EFA and CFA results.

EFA										CFA	
	Factors							Explained variance (%)	Cronbach’s alpha	Standardized loading	AVE
	1	2	3	4	5	6	7				
**R6**	0.886							62.3%	0.955	0.863	0.674
**R8**	0.884									0.890	
**R7**	0.878									0.899	
**R5**	0.832									0.829	
**R3**	0.804									0.826	
**R2**	0.789									0.799	
**R11**	0.784									0.710	
**R9**	0.780									0.853	
**R10**	0.779									0.887	
**R4**	0.732									0.795	
**R1**	0.429									0.637	
**L5**		0.884						68.0%	0.938	0.888	0.727
**L3**		0.884								0.887	
**L1**		0.879								0.880	
**L2**		0.862								0.849	
**L4**		0.793								0.856	
**L6**		0.611								0.749	
**O4**			0.898					56.1%	0.933	0.789	0.699
**O5**			0.879							0.848	
**O6**			0.759							0.871	
**O3**			0.759							0.843	
**O2**			0.626							0.850	
**O1**			0.491							0.815	
**MAKM2**				0.977				63.8%	0.959	0.877	0.799
**MAKM4**				0.873						0.913	
**MAKM1**				0.807						0.889	
**MAKM5**				0.785						0.888	
**MAKM3**				0.716						0.901	
**MAKM6**				0.574						0.810*	
**C5**					0.918			46.6%	0.923	0.856	0.741
**C4**					0.898					0.871	
**C3**					0.698					0.726*	
**C6**					0.627					0.856	
**C2**					0.393					0.854*	
**C1**					0.332					0.854*	
**W6**						0.826		50.6%	0.935	0.900	0.710
**W4**						0.772				0.837	
**W3**						0.744				0.853	
**W2**						0.736				0.795	
**W5**						0.626				0.869	
**W1**						0.521				0.797	
**S4**							0.916	41.1%	0.927	0.912	0.757
**S3**							0.624			0.847	
**S2**							0.592			0.834*	
**S5**							0.582			0.850	
**S1**							0.372			0.778*	

* Items removed in the CFA.

### Results of the Confirmatory Factor Analysis (CFA)

The validity of a measurement model depends on establishing acceptable levels of goodness of fit and finding specific evidence of construct validity. Hair et al. [35] and Kline [52] claim that the use of three to four indices usually provides adequate evidence of model fit. Some of these indices include the χ2 statistic, the root mean square error of approximation (RMSEA) index, the comparative fit index (CFI) or the Tucker-Lewis index (TLI), and the standardized root mean residual (SRMR) index. The Confirmatory Factor Analysis (CFA) was performed on software program SPSS Amos® 23. As Table 7 displays, we computed 12 indices, nine of which showed acceptable values.

**Table 7:** Goodness of fit tests and results.

Goodness-of-fit statistics	Measurement model	Research model and hypotheses	Structural model results	Recommended values
**χ2/df**	2.61	2.62	2.62	3 or less [59].
**Goodness-of-fit index (GFI)**	0.8477	0.8473	0.8468	Close to .90 [60].
**Normed Fit Index (NFI)**	0.9191	0.9186	0.9183	Close to .90 or .95 reflects a good model fit [60].
**CFI**	0.9483	0.9479	0.9478	Greater than 0.9 [35,60]-
**TLI**	0.9439	0.9437	0.9439	Greater than 0.9 [35,60].
**Root-mean residual (RMR)**	0.0316	0.0328	0.0331	0.05 or less [61].
**RMSEA**	0.0543	0.0544	0.0543	Lower than 0.08 [62].
**Parsimony goodness-of-fit index (PGFI)**	0.7433	0.7460	0.7497	Greater than 0.5 [61].
**Parsimony normed fit index (PNFI)**	0.8472	0.8503	0.8547	Greater than 0.5 [61].
**Akaike’s information criterion (CAIC)**	2617.75	2608.31	2585.85	<Saturated model and independent model [61].
**CAIC for saturated model**	5991.15	5991.15	5991.15	
**CAIC for independent model**	23528.24	23528.24	23528.24	

#### Construct validity

Construct validity comprises convergent validity and discriminant validity. Convergent validity determines the degree to which multiple items coincide to measure the same concept [63]. To measure convergent validity, we calculated the Average Variance Extracted (AVE) of each construct and compared it with that construct’s correlation with the other constructs. In cases when the AVE was greater than the construct’s correlation with the other constructs, convergent validity was confirmed. In this sense, AVE values greater than 0.5 are usually indicators of good convergent validity [35]. Table 6 lists the AVE value of each construct, and since all the values are greater than 0.5, we concluded that the theoretical model has enough convergent validity.

Discriminant validity measures whether one construct is different from another construct [64]. We measured discriminant validity as suggested by Fornell and Larcker [65]; that is, if the square root of the AVE of a construct was greater than its correlation coefficient with another construct, the two constructs were considered to be different from each other. To ensure discriminant validity in our data, we removed some items from the analysis, thus making the values of the correlation coefficients lower than the square root of the AVEs. The results of the discriminant validity test are summarized in Table 8 and indicate that all the constructs have discriminant validity.

**Table 8:** Bivariate correlation between constructs and square root of AVEs.

Construct	MAKM	Results	Workforce	Operations	Strategy	Customers	Leadership
**MAKM**	**0.894**^**a**^						
**Results**	0.795	**0.821**^**a**^					
**Workforce**	0.842	0.788	**0.843**^**a**^				
**Operations**	0.764	0.744	0.827	**0.836**^**a**^			
**Strategy**	0.850	0.771	0.827	0.783	**0.870**^**a**^		
**Customers**	0.823	0.728	0.724	0.675	0.828	**0.861**^**a**^	
**Leadership**	0.717	0.723	0.735	0.699	0.814	0.718	**0.853**^**a**^

^a^ square root of AVE

### Model assessment and hypothesis testing

SEM is a statistical technique rather confirmatory in testing research hypotheses representing relationships between latent variables or constructs [61]. In addition, SEM allows for the simultaneous analysis of a series of causal relationships between multiple constructs [63]. We tested the model depicted in Fig 2 using the Maximum Likelihood estimation method and software program IBM SPSS Amos 23. The results from the model fit test are summarized in Table 7 and confirm that the model fits the data well. Therefore, the hypotheses can be analyzed.

### Evaluating hypothesized relationships using SEM

Table 9 lists the results from the hypotheses test, including the standardized regression coefficients, the critical ratio (CR), and the probability value (P). Kline [52] claims that CR values greater than 1.96 indicate statistical significance in relationships. Thus, according to our results, 14 of the 18 hypotheses represent statistically significant relationships. The non-significant relationships comprised Leadership-Customers, Workforce-Customers, MAKM-Operations, and Customers-Operations.

**Table 9:** Hypothesis test results.

Path			Standardized Regression Weights Estimates	SE	CR	P	Results
MAKM	←	Leadership	0.721	0.048	16.00	***	Supported
Strategy	←	Leadership	0.440	0.045	10.56	***	Supported
Strategy	←	MAKM	0.534	0.041	13.18	***	Supported
Customers	←	Leadership	0.070	0.061	1.22	0.2236	Not supported
Customers	←	MAKM	0.416	0.064	6.50	***	Supported
Customers	←	Strategy	0.419	0.086	4.81	***	Supported
Workforce	←	MAKM	0.509	0.062	7.88	***	Supported
Workforce	←	Strategy	0.484	0.067	6.88	***	Supported
Workforce	←	Customers	-0.096	0.060	-1.54	0.1238	Not supported
Operations	←	MAKM	0.099	0.077	1.35	0.1757	Not supported
Operations	←	Strategy	0.311	0.084	3.84	***	Supported
Operations	←	Customers	-0.029	0.069	-0.44	0.6589	Not supported
Operations	←	Workforce	0.504	0.073	7.50	***	Supported
Results	←	Leadership	0.181	0.037	4.00	***	Supported
Results	←	MAKM	0.267	0.054	3.84	***	Supported
Results	←	Workforce	0.221	0.054	3.29	***	Supported
Results	←	Operations	0.155	0.040	2.89	0.004**	Supported
Results	←	Customers	0.114	0.044	2.03	0.042*	Supported

*** Significant at a 0.001 level

** significant at a 0.01 level

* significant at a 0.05 level

SE: standard error; CR: critical ratio.

**Fig 4:**
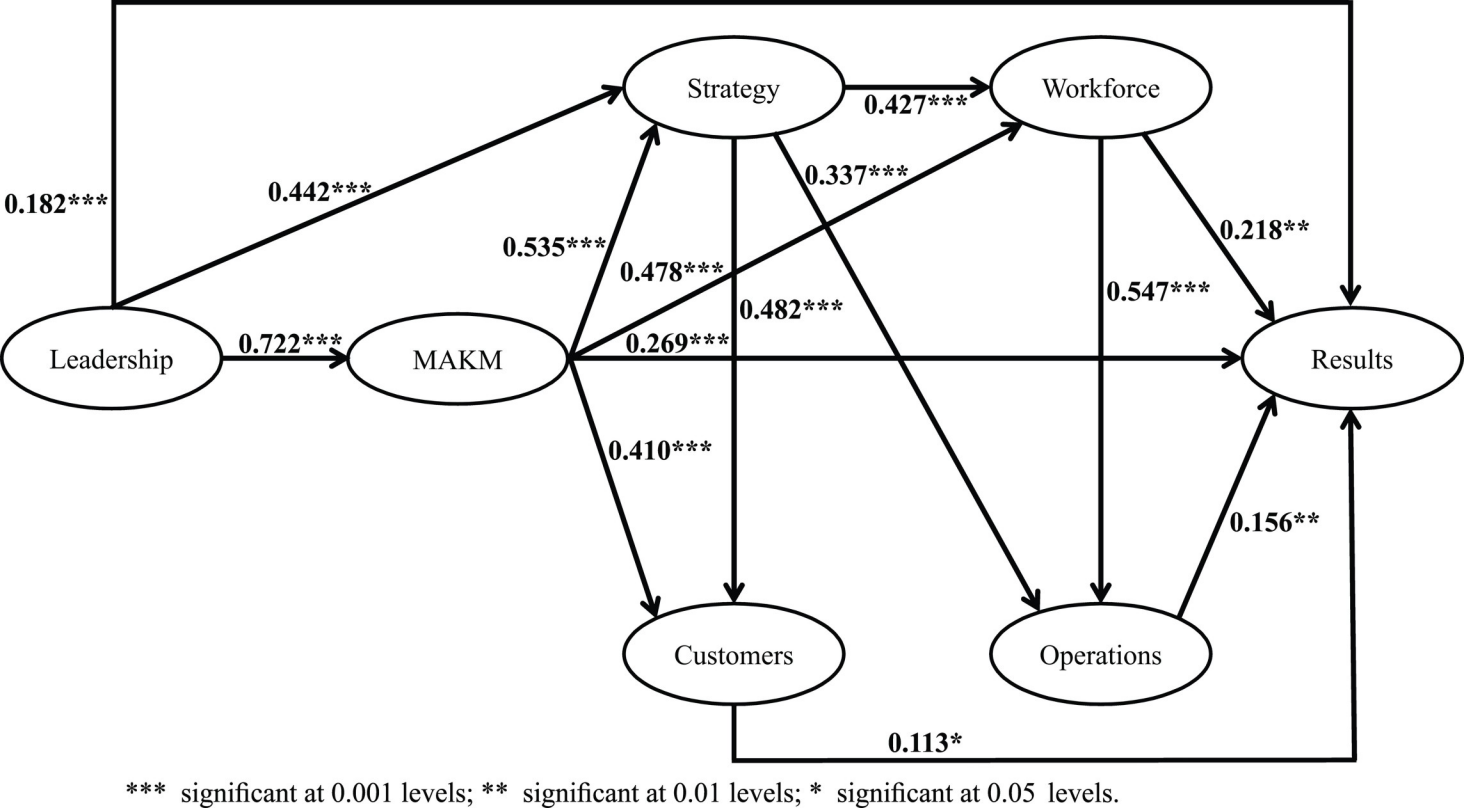
Final structural model.

Fig 4 depicts the final model, once the four non-significant hypotheses were removed. The model fit indices of the final model are listed in Table 7.

## Discussions

This work explores and tests the relationships among the constructs of leadership, strategy, clients, MAKM, workforce, and operations to propose a model that serves as the basis for conducting IPs in Mexican public HEIs. In the field of education, the BPEP is internationally recognized as a tool for attaining excellence. It is a program with a solid structure, where top management initiates any improvement action, and it is followed by the other organizational elements and factors, which together interact and pursue a common outcome. According to our findings, leadership has a positive impact—either direct or indirect—on all the other constructs, thus making it possible to obtain the expected benefits from the implemented IPs. This finding is consistent with the general theory that "leadership drives the system which creates results" [34]. Likewise, it coincides with what was reported by Lu, Laux and Antony [66], who said that an adaptive leadership structure can guarantee a successful adoption of the techniques as the basis for this continuous improvement and help foster an environment that recognizes and maintains the achievements. In other words, HEIs willing to implement IPs must focus on managerial leadership, since it is the quality promoter and controller. According to Flynn and Saladin [38], senior management must create an adequate infrastructure for quality management. Thus, the benefits of IPs would apply to all those involved in the organization, such as students, professors, and society in general.

This study demonstrates that senior management at all levels is responsible for initiating IPs. As Badri et al. [8] point out, HEI leaders have the ability and significant influence to make changes in the educational system; hence, they must guide each strategy that seeks to achieve excellence in their institutions. Likewise, [39] concludes that leadership is the force that leads to all the elements of the quality management system. This does not mean that the other system dimensions are not important but rather that leadership guides their development so that each of them contributes with an essential part to reach the desired results. Leadership focuses on developing strategic goals, implementing a suitable information and communication system, integrating the workforce, and improving processes to meet customer needs and attain the desired outcomes. Finally, our model found that leadership has the strongest impact (i.e., 0.722) on MAKM, which is consistent with what Badri et al.[8] showed. Such results imply that the surveyed leaders recognize that a solid information system is important for objective decision making. In the same way, MAKM has a positive effect on strategies, customers, workforce and operations. This result is consistent with that obtained by Winn and Cameron [40], in which the information system is necessary for strategic planning involving human resources, operations and the client. Likewise,[67] conclude that the information system plays a critical role in the quality management system, since we are in the information age.

The strategy dimension is a management activity that establishes goals considering customer demands, staff training, and continuous improvement [8,36]; hence, it has direct effects on an organization’s operations, workforce, and customers. Process quality improvement is achieved through a workforce that follows the HEI’s strategies, thus maintaining a high-performance workplace focused on the students, which justifies the direct relationship with both operations and results. These findings are consistent with those of [68], who concluded that when the university establishes a quality control-oriented workplace, it is more likely that academic staff will be satisfied and therefore work constructively for the cause of organizational success.

On the other hand, the customer dimension prioritizes the measurement of the satisfaction of students, staff, and society in general, and it listens to them through result indicators, which is consistent with the findings reported by Antony et al. [69], such that in HEIs, it is important to change the way customers are cared for to provide a world-class experience. For the operations criterion, it is the means through which an HEI develops the strategies responding to the clients' requirements to obtain the planned results. Similarly, [67] consider in their findings that an important factor in service organizations such as HEIs is that the customer is extremely important. The strategy criterion does not have a direct effect on the results; however, it indirectly contributes to obtaining the results of HEIs because it directly and positively impacts workforce and operations, which have a direct effect on the results. The motivation for employees to participate in quality planning helps them to perform their tasks better [70]. In addition, the participation of employees in the operationalization of the strategies creates the necessary conditions for the continuous improvement of the processes [71,72]. Our proposed model shows that excellent results are obtained through the relationships among the other dimensions together.

## Conclusions

In summary, the main contributions of the study are highlighted. First, the results of the EFA confirm the feasibility of the factor analysis, since the results shown in Table 6 meet the indicators recommended in the literature (the KMO value must be greater than 0.90, and the p-value for Bartlett’s sphericity test must be lower than 0.01). Subsequently, the results of the CFA validate the measurement model, and Table 7 shows the values of the model adjustment indices that satisfy the values recommended by the literature. Finally, in the structural model, 18 hypotheses were verified that were related to the interrelations between the quality dimensions of the BPEP model and to explain the effects of these quality dimensions on the results. Ultimately, only four proposed relationships were not significant.

Our findings indicate that leadership is the starting point for conducting IPs and obtaining the planned results. Strong leadership promotes the participation and integration of those involved in each model dimension. In addition, our model reaffirms the importance of information systems, which are the means of collecting and analyzing information to be shared across the dimensions and used to predict problems and make improvement decisions. The role of the strategy, workforce, and operations dimensions is to plan and make all the improvements to turn them into favorable results, with customer satisfaction as the ultimate goal. Finally, we also found that the strongest relationships in the model include leadership-MAKM (0.722) and workforce-operations (0.547). Hence, HEI leaders must pay close attention to these relations when implementing IPs. Therefore, it can be said that the BPEP is a model that has well-defined criteria, and the findings highlight the significant relationships among these criteria, as well as the role they play in improving the quality of the processes. Then, it can be concluded that to increase the chances of obtaining favorable results, there must be a form of leadership committed to improvement, a well-established measurement system in the institution, a clearly defined strategy and a workforce responsible for the execution of IPs.

HEIs face intense competition every day due to the globalization of the business world, which demands that quality knowledge and skills meet the challenges demanded by employers, as well as detect and meet unmet needs through the entrepreneurship of their own business. Considering the need for and importance of improving the quality of administrative processes, in addition to teaching and learning, this research was conducted to propose a model that provides a structure for the realization of IP in the IES based on BPEP structure. We relied on the SEM technique to validate the model and found that the model can serve as a guide for all HEIs interested in improving the quality of their processes through IPs. This is the main contribution of our research to the literature on continuous improvement in educational institutions. Moreover, it is worth mentioning that similar research in the Latin American region is scarce; hence, the contribution is greater.

## Limitations and future research

This study has some limitations that may direct future research. Mainly, potential confounding variables (including different departments, faculties and schools, etc.) were not controlled for in our study and from this fact some limitations and possible future work emerge. First, the model was developed and tested with data gathered from Mexican universities. Second, our model was purposely designed for public universities and may not be suitable for private universities in its original version. Third, the study is cross-sectional, which means that the data were analyzed at specific points in time. With the previous information, future work can be developed to overcome each of the limitations. For the first limitation, a line of future research may be the validation of the model in other countries, which implies that its applications must be preceded by a thorough review of the literature to address cultural and regional idiosyncrasies. A second line of future research that corresponds to the second limitation may be the application of the model in private universities to examine whether the model fits its operations, which would provide additional validation of the model proposed in this study, since, as mentioned by [73], public and private universities can learn from each other regarding different types of organizational culture. As a third line of future work, a longitudinal study would be advisable to compare the different stages of IP adoption over a period of time. In addition, a line of future research could be the application of the model at different levels of education, with respect to the necessary adjustments, to adapt the model to the organizational structures of these institutions.

From the ideas expressed in the previous paragraph, it should be emphasized that although the authors of the present work consider that the resultant model could be applied in other countries and probably, even in the context of other service organizations outside the education sector, some cautions should be exercised before the generalization of the results reported here and greater care in the applicability of this model should be taken when the situation becomes more different from the conditions and the environment in which this model was validated.

## Disclosure statement

The authors declare no potential conflicts of interest with respect to the publication of this research.

## Supporting information

S1 Table Correlations matrix. (XLSX)

S2 Table Collinearity statistics. (XLSX)

S3 Table Assessment of normality. (XLSX)
